# Fluocinolone acetonide implant in diabetic macular edema: sustained benefits and effects of following prior therapy

**DOI:** 10.1186/s40942-026-00861-y

**Published:** 2026-04-26

**Authors:** Philipp Necpal, Klemens P. Kaiser, Ron C. Kroener, Pankaj Singh

**Affiliations:** 1 Department of Ophthalmology, Goethe-University, Frankfurt, Germany; 2 Division Chief Vitreoretinal Surgery, Department of Ophthalmology, Goethe-University Frankfurt, Theodor-Stern-Kai 7, 60590 Frankfurt am Main, Germany

## Abstract

**Purpose:**

To (1) evaluate long-term real-world anatomical and functional outcomes after implantation of a sustained-release fluocinolone acetonide (FAc) implant in eyes with diabetic macular edema (DME) and (2) to assess the influence of prior anti–vascular endothelial growth factor (anti-VEGF) and corticosteroid exposure on treatment response and durability.

**Methods:**

This retrospective, single-center study included 74 eyes from 47 DME patients treated with intravitreal FAc implant. Best-corrected visual acuity (BCVA), central retinal thickness (CRT), intraocular pressure (IOP), prior treatment burden, and need for additional intravitreal therapy were evaluated over a median follow-up of 40 months. Baseline changes were analysed using paired statistical testing. Spearman correlations assessed associations between prior treatment exposure and clinical outcomes.

**Results:**

FAc implantation significantly reduced CRT (median −75.5 µm; p<0.001). Eyes receiving fewer than six prior anti-VEGF injections demonstrated greater CRT reduction (−93 µm; p<0.001) than eyes receiving six or more injections (−65 µm; p=0.015). BCVA improved modestly overall (+3.95 ETDRS letters; p=0.037), with greater gains in less pretreated eyes (+7.53 letters; p=0.002) compared to eyes with ≥6 prior injections (+0.0 letters; p=0.492). Higher prior anti-VEGF burden correlated with shorter treatment durability (ρ = −0.35; p<0.003) and increased need for supplemental therapy (ρ = 0.30; p<0.001).

**Conclusions:**

FAc implantation provides sustained anatomical improvement and modest functional benefit in DME, particularly when introduced earlier in the treatment course. Earlier integration of long-acting corticosteroid therapy may enhance durability and reduce cumulative anti-VEGF burden.

## Background

Diabetic macular edema (DME) is a major cause of visual impairment among adults in developed countries [1]. It is a multifactorial disease that directly compromises central vision, affecting approximately 12% of individuals with Type 1 diabetes and 28% of those with Type 2 diabetes within nine years of diagnosis [2]. The treatment options of DME were limited to focal or grid macular laser photocoagulation and periocular or intravitreal administration of short-acting corticosteroids, including the off-label use of triamcinolone acetonide [3, 4]. Anti-vascular endothelial growth factor (anti-VEGF) therapies with agents such as ranibizumab, aflibercept, faricimab and brolucizumab have revolutionised the management of DME and are now considered first-line treatments in DME [5]. However, up to 40% of patients demonstrate a suboptimal response to anti-VEGF therapy [6]. In these patients, intravitreal corticosteroids provide a valuable alternative as they target both anti-VEGF and inflammatory pathways. Treatment options include sustained-release dexamethasone (OZURDEX^→^, Allergan Inc. Irvine, California) and fluocinolone acetonide implant (FAc; ILUVIEN^Ⓡ^, 0.19 mg intravitreal implant in applicator, Alimera Sciences Europe Ltd., Dublin, Ireland) [7–9]. 

The FAc implant has been approved in several European countries for the treatment of DME considered insufficiently responsive to available therapies [9, 10]. 

Over the last decade, randomised controlled trials [11–13] and real-world clinical practice studies [14–18] have assessed the efficacy and safety of the FAc implant, demonstrating a favourable and reproducible efficacy and safety profile. Notably, a review including data from 22 real world studies reported a mean visual acuity gain of + 8.7 letters (range: 0.4 to 18.8. letters, median: + 8.0 letters) and a maximum reduction in CRT of -34.3% (range: -10.7 to -55.8%, median: -36.2.%) from baseline [19]. Importantly, treatment history appears to influence subsequent outcomes with FAc therapy. Real-world evidence suggests that not only the extent of prior treatment but also the timing of switching to FAc therapy influences outcome [15, 19–22]. In patients with suboptimal response to anti-VEGF or short-acting steroids, an earlier switch to FAc has been associated with more stable anatomical control and reduced need for additional interventions [23]. As these findings suggest that treatment burden and disease chronicity should be considered when interpreting response to FAc therapy, this study evaluates real-world anatomical and functional outcomes following FAc implantation in patients with DME. It also assesses the influence of prior anti-VEGF and steroid treatments on outcomes.

## Methods

This retrospective, consecutive case series was conducted at the Department of Ophthalmology, Goethe University Frankfurt am Main, Germany, a single tertiary referral center. Medical records from patients with a history of DME who were treated with the FAc implant between November 2014 and July 2025 were reviewed. Data from these medical records was extracted and included: ophthalmic imaging examinations (optical coherence tomography and fluorescein angiography), surgical reports, medication and intravitreal injection records, as well as clinical findings and longitudinal documentation (e.g., physician correspondence and standardized follow-up questionnaires). Best-corrected visual acuity (BCVA), measured in ETDRS letters, was assessed in a clinical setting using either autorefraction or subjective refraction, according to routine clinical practice. Postoperative outcome measurements were obtained at least 6 months after FAc implantation. Institutional review board approval was obtained from the Ethics Committee of Goethe University Frankfurt am Main, Germany (approval number 2025–2683), and the study was conducted in accordance with the tenets of the Declaration of Helsinki. Given the retrospective study design, the requirement for informed consent was waived by the Ethics Committee.

### Inclusion and exclusion criteria

Only patients with a clinically confirmed diagnosis of DME who received an intravitreal FAc as part of routine clinical care were included. Inclusion criteria comprised a confirmed diagnosis of DME, implantation of the FAc implant within the predefined observation period, and availability of complete and relevant follow-up data for a minimum of 12 months. In this study patients were included presenting with phakic and pseudophakic eyes. Patients were excluded if follow-up data were missing or incomplete, or if concomitant ocular comorbidities with a substantial potential impact on visual acuity were present, including age-related macular degeneration, retinal vein occlusion, high myopia, choroidal neovascularisation related to high myopia, ocular hypertension or glaucoma.

### Study endpoints

Baseline demographic and clinical characteristics were collected, including age, sex, and a confirmed diagnosis of diabetes mellitus (disease duration and HbA1c levels). Prior ocular treatments were recorded, including retinal laser photocoagulation or cryocoagulation, pars plana vitrectomy, intravitreal anti-VEGF therapy, and intravitreal corticosteroid injections. Baseline lens status (phakic or pseudophakic) and intraocular pressure, measured without the use of intraocular pressure-lowering medication, were also documented.

### Main outcome measures

The primary outcome measures were changes from baseline in BCVA and CRT, as assessed by optical coherence tomography using the ETDRS grid following initiation of treatment with the FAc implant. Secondary outcome measures included the frequency and timing of additional intravitreal injections, changes in intraocular pressure (IOP), the occurrence of treatment-related adverse events or complications, and the need for additional therapeutic interventions during the follow-up period, including cryocoagulation, laser photocoagulation or pars plana vitrectomy.

### Statistical analysis

Statistical analyses were primarily descriptive and performed using GraphPad Prism version 8 (GraphPad Software, San Diego, CA, USA). Continuous variables (e.g., BCVA, CRT, and IOP) are reported as mean ± standard deviation or median with interquartile range (IQR), depending on normal distribution, which was assessed using the Shapiro-Wilk test. Changes from baseline over time were analyzed using paired statistical tests (paired t-test or Wilcoxon signed-rank test, depending on data distribution). Statistical analyses were performed at the eye level without the use of mixed-effects models to account for inter-eye correlation. Categorical variables are presented as absolute counts (*n*) and percentages. A two-sided *p*-value < 0.05 was considered statistically significant.

## Results

The baseline demographic and clinical characteristics of the 74 eyes from 47 patients included in this study are summarized in Table 1. Overall, the cohort had a median age of 63 years a median follow-up of 40 months moderate visual impairment and increased central retinal thickness at baseline, and was characterized by a high prior treatment burden, with the majority of eyes having received previous intravitreal therapies.

**Table 1:** Baseline characteristics of 74 eyes of 47 patients included in the analysis

Characteristic	
**Age** (median (IQR), [range])	*63 (55.00–72.00 [23–88])*
**Sex** (female; n; %)	*23(48%)*
**Right Eyes** (n; %)	34 (46%)
**BCVA** (ETDRS; median (IQR), [range])	*58.00 (35.00-66.25 [0.00–80.00])*
**Follow-up** (months; median (IQR), [range])	*40 (20.50–81.00 [6–118])*
**Lens Status**	
*** Phakic eyes*** *(n; %)*	*35 (47%)*
*** Pseudophakic eyes*** *(n; %)*	*39 (53%)*
**HbA1c** (%; median (IQR), [range])	*7.20 (6.40–7.65 [0.00–11.00])*
**Central retinal thickness** (µm; median (IQR), [range])	*421 (327.5–509.5 [186–824])*
**Pre-treatments**	
*** Vitrectomy (n; %)***	*8 (10%)*
*** Peripheral Argon-laser koagulation (n; %)***	*39 (53%)*
*** Any intravitreal anti-VEGF (n***,*** %)***	*33 (45%)*
*** Median number of anti-VEGF treatments*** *(median (IQR)*,* [range])*	*0 (0–6 [0–28])*
*** Any intravitreal corticosteroid (n***,*** %)***	*63 (85%)*
*** Median number of steroid treatments (****median (IQR)*,* [range])*	*2 (1–3 [0–9])*

BCVA = best-corrected visual acuity; n = number; SD = standard deviation

**Fig. 1:**
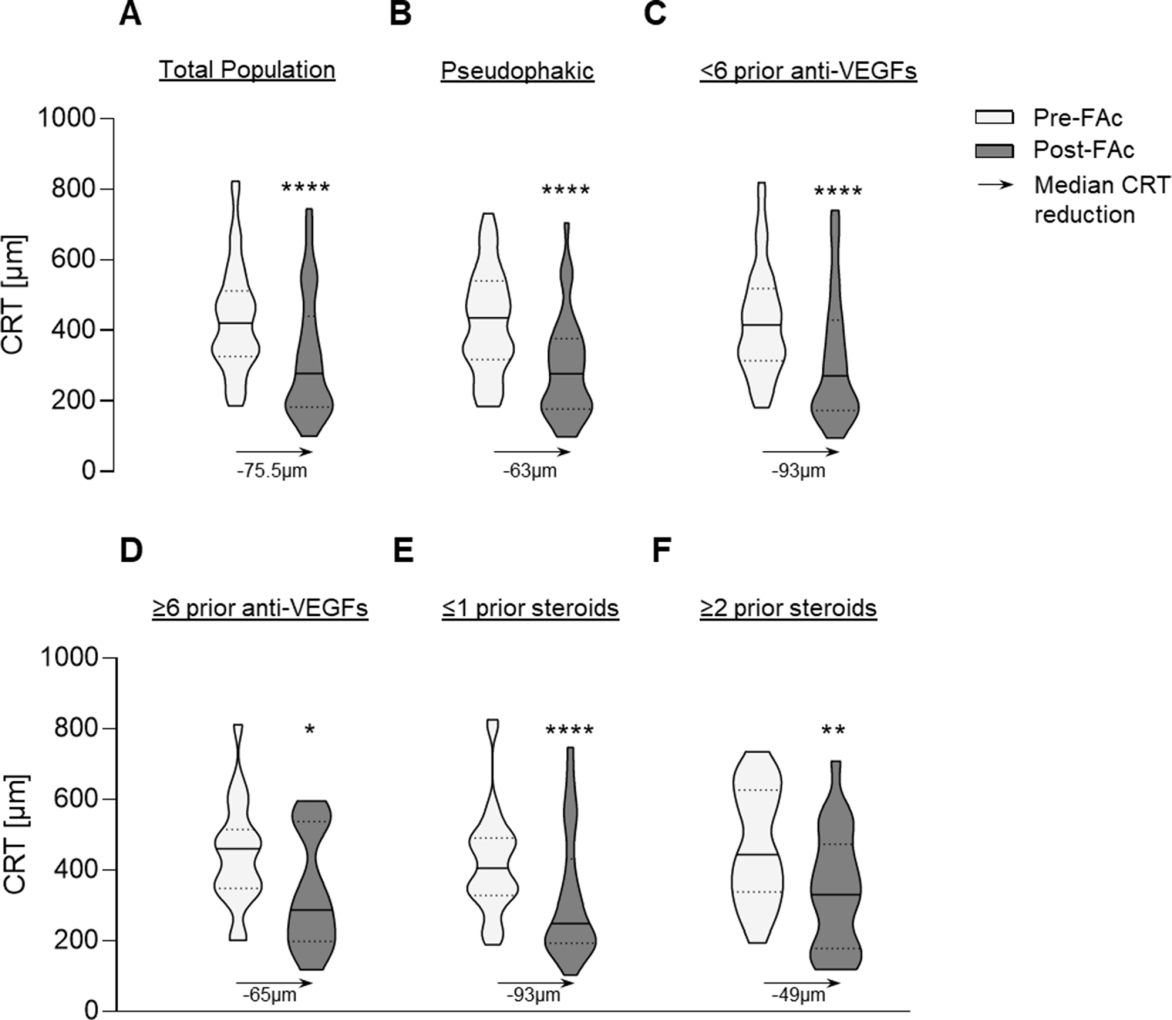
Violin plots illustrating the changes in central retinal thickness (CRT) with median of differences (µm) across different conditions: (**A**) before and after fluocinolone acetonide (FAc) implantation (*n* = 68; *p* < 0.0001); (**B**) in pseudophakic patients before and after FAc implantation (*n* = 35; *p* < 0.0001); (**C**) prior to ≤ 6 anti-VEGF treatments (*n* = 49; *p* < 0.0001); (**D**) following six or more anti-VEGF injections (*n* = 19; *p* = 0.0145); (**E**) after fewer than one steroid treatment (≤ 1 steroid; *n* = 47; *p* < 0.0001); and (**F**) after more than two steroid treatments (≥ 2 steroids; *n* = 21; *p* < 0.003). **p < 0.01, ***p < 0.001, ****p < 0.0001

Changes in CRT following FAc implantation are summarized in Fig. 1A-F in the overall cohort (Fig. 1A), CRT decreased significantly from baseline to follow-up (Wilcoxon matched-pairs signed-rank test; median change, -75.5 μm; *n* = 68; *p* < 0.001), with moderate but significant pairing (Spearman’s ρ = 0.39; *p* < 0.001).

A significant CRT reduction was also observed in pseudophakic eyes (Fig. 1B; median change, -63 μm; *n* = 35; *p* < 0.001), although pairing effectiveness did not reach statistical significance (ρ = 0.22; *p* = 0.097). Eyes receiving fewer than six prior anti-VEGF injections (Fig. 1C) demonstrated a pronounced decrease in CRT (median change, -93 μm; *n* = 49; *p* < 0.0001), with significant pairing effectiveness (ρ = 0.41; *p* = 0.002). In contrast, receiving six or more prior anti-VEGFs (Fig. 1D) also showed a significant reduction in CRT, although the magnitude of reduction was less marked (median change, -65 μm; *n* = 19; *p* = 0.0145) and pairing effectiveness was not statistically significant (ρ = 0.38; *p* = 0.055).

Eyes with fewer than one prior intravitreal corticosteroid treatment (Fig. 1E) exhibited a marked CRT reduction (median change, -93 μm; *n* = 47; *p* < 0.001). The majority of prior treatments included dexamethasone injections and in a minority of cases intravitreal triamcinolone was applied. In contrast, eyes with prior corticosteroid exposure (Fig. 1F) showed a smaller but still significant decrease in CRT (median change, -49 μm; *n* = 21; *p* = 0.003), pairing not significant (ρ = 0.29; *p* = 0.099).

**Fig. 2:**
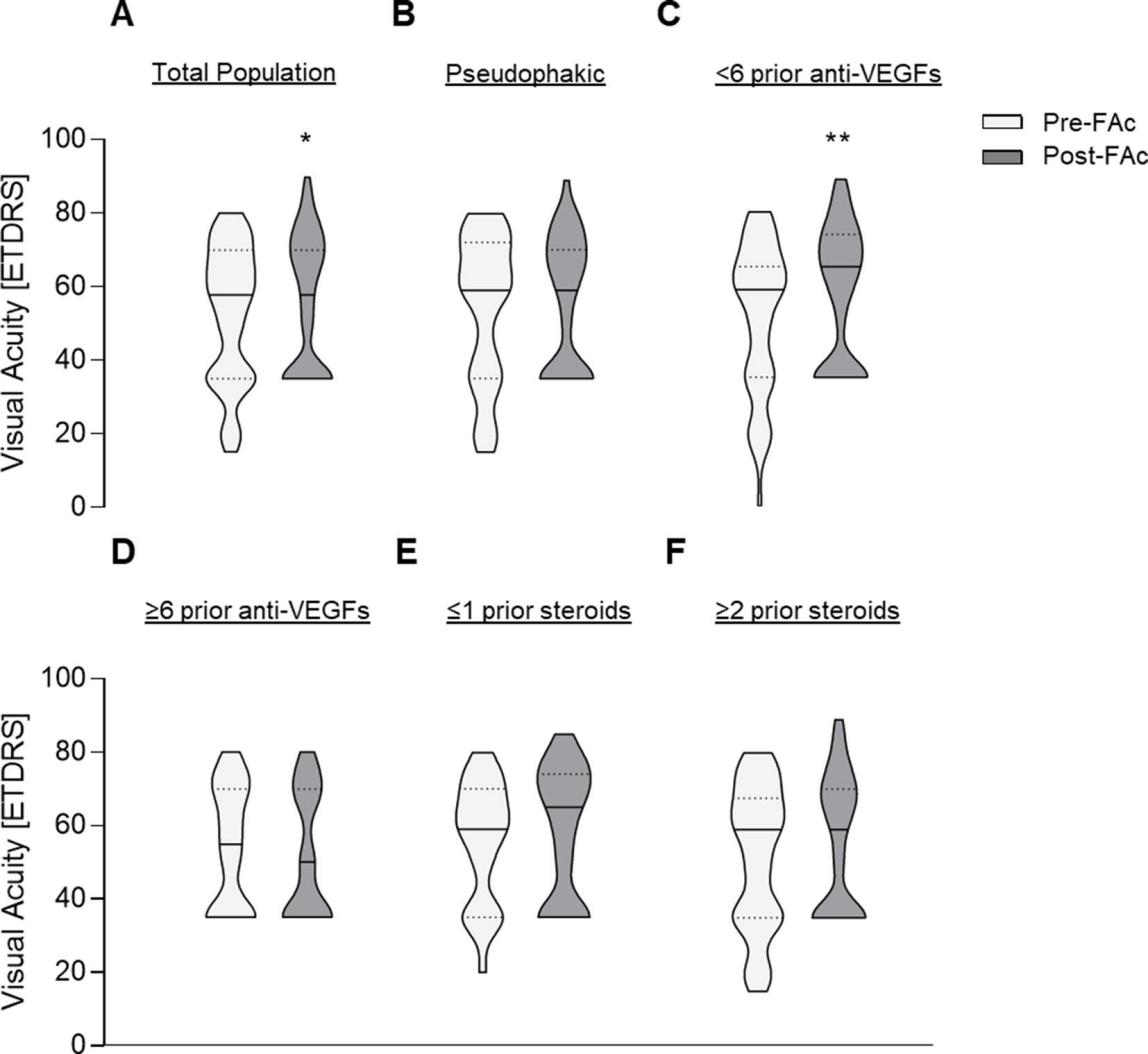
Violin plots illustrating the changes in best-corrected visual acuity (BCVA) across different subgroups: (**A**) before and after fluocinolone acetonide (FAc) implantation (*n* = 70; *p* = 0.026); (**B**) in pseudophakic patients before and after FAc implantation (*n* = 37; *p* = 0.271); (**C**) in patients with fewer than six prior anti-VEGF injections (*n* = 50; *p* = 0.002); (**D**) changes in patients with six or more prior anti-VEGF therapies (*n* = 21; *p* = 0.49); (**E**) changes in patients with fewer than one prior corticosteroid treatment (≤ 1 corticosteroid; *n* = 25; *p* = 0.236); and (**F**) changes in patients with more than two prior corticosteroid treatments (≥ 2 corticosteroids; *n* = 45; *p* = 0.061). *p < 0.05; **p < 0.01

Changes in BCVA (ETDRS letters) before and after FAc implantation in the overall cohort and predefined subgroups are shown in Figs. 2A-F. In the overall study population (Fig. 2A), BCVA improved significantly following FAc implantation (Wilcoxon matched-pairs signed-rank test; *p* = 0.037), with a median improvement of 3.95 ETDRS. Pairing effectiveness was strong and highly significant (ρ = 0.57; *p* < 0.001), supporting robust within-subject comparisons.

No significant change in BCVA was observed in pseudophakic eyes (Fig. 2B; *p* = 0.271; median change, 0 ETDRS), despite strong and significant pairing effectiveness (ρ = 0.64; *p* < 0.001). Eyes receiving fewer than six prior anti-VEGF treatments demonstrated a significant improvement in BCVA after FAc implantation (Fig. 2C; *p* = 0.002; median change, + 7.53 ETDRS), accompanied by strong pairing effectiveness (ρ = 0.55; *p* < 0.001). In contrast, eyes with prior exposure to six or more anti-VEGF therapies did not show a significant BCVA change (Fig. 2D; *p* = 0.492; median change, 0 ETDRS), although pairing effectiveness remained strong (ρ = 0.67; *p* < 0.001), indicating interindividual variability in treatment response.

BCVA changes were not statistically significant in eyes with fewer than one prior intravitreal corticosteroid treatment (Fig. 2E; *p* = 0.236; median change, 0 ETDRS) or in eyes with more than two prior corticosteroid injections (Fig. 2F; *p* = 0.09; median change, + 5.0 ETDRS). In both subgroups, pairing effectiveness was moderate to strong and statistically significant (ρ ≥ 0.48; *p* < 0.001).

**Fig. 3:**
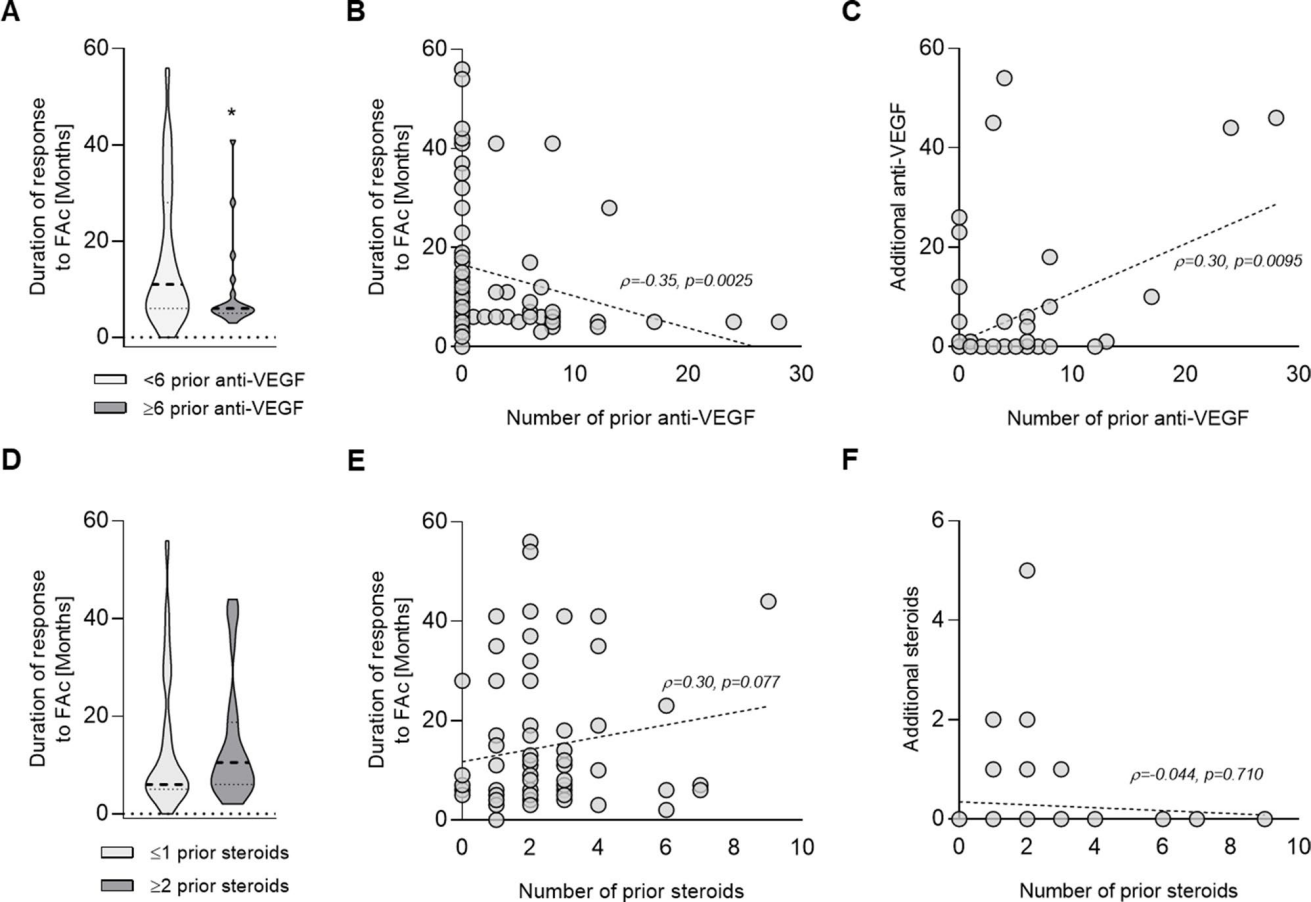
Associations between prior treatments and the duration and need for additional therapy following fluocinolone acetonide (FAc) implantation. (**A**) Duration of the therapeutic effect of FAc implantation in relation to prior anti-VEGF treatments (< 6 anti-VEGFs, *n* = 53 vs. ≥6 anti-VEGF, *n* = 21; *p* = 0.015). (**B**) Correlation between the number of prior anti-VEGF treatments and the duration of FAc efficacy. (**C**) Association between prior anti-VEGF treatment burden and the requirement for additional anti-VEGF therapy after FAc implantation. (**D**) Comparison of treatment effect duration in patients with fewer than one versus more than two prior corticosteroid treatments (≤ 1 vs. ≥2 steroids; *p* = 0.461). (**E**) Correlation between the number of previous corticosteroid treatments and the duration of FAc effect. (**F**) Association between prior corticosteroid exposure and the need for additional corticosteroid treatment following FAc implantation. *p < 0.05

The duration of response to FAc was analyzed with respect to prior intravitreal injections of anti-VEGF and intravitreal corticosteroid agents. Eyes receiving fewer than six prior anti-VEGF injections demonstrated a significantly longer treatment-free interval after FAc (11.0 months; *n* = 53) than heavily pretreated eyes with anti-VEGF (6.0 months; *n* = 21; Mann-Whitney U = 357; two-tailed *p* = 0.015) (Fig. 3A). The median difference was − 11 months (Hodges-Lehmann estimate − 3 months), indicating reduced treatment durability with the extent of prior anti-VEGF injections. Correlation analysis demonstrated a significant negative association between the number of prior anti-VEGF injections and duration of effect (ρ=-0.35; 95% CI, -0.54 to -0.12; *p* = 0.0025; *n* = 74), suggesting progressively shorter treatment effects with increasing cumulative anti-VEGF exposure (Fig. 3B).

A significant positive correlation was observed between prior and additional anti-VEGF injections (ρ = 0.30; 95% CI, 0.07 to 0.50; *p* = 0.0095; *n* = 74), indicating that eyes with greater prior anti-VEGF exposure were more likely to require further anti-VEGF therapy after FAc implantation (Fig. 3C).

In contrast to anti-VEGF injections, prior corticosteroid treatment did not significantly influence the duration of response to FAc. No clinically meaningful difference in treatment duration was observed between eyes with fewer than one prior corticosteroid treatment (6.0 months; *n* = 50) and those with ≥ 2 prior injections (10.5 months; *n* = 24; Mann–Whitney U = 536; two-tailed *p* = 0.461; Hodges–Lehmann median difference = 1 unit) (Fig. 3D). Consistently, no significant correlation was observed between the number of prior corticosteroid treatments and either duration of response to FAc (ρ = 0.21; 95% CI, -0.3 to 0.42; *p* = 0.077; *n* = 74; Fig. 3E) or the need for additional corticosteroid therapy during follow-up (ρ=-0.044; 95% CI, -0.28 to 0.19; *p* = 0.710; *n* = 74; Fig. 3F).

A correlation analysis between changes in ΔCRT and ΔBCVA revealed no statistically significant association in the overall cohort (ρ=-0.13, 95% CI, -0.37 to 0.13; *p* = 0.32; *n* = 64). Despite a slight trend towards greater visual improvement with increasing CRT reduction, this did not reach statistical significance in any group.In total, 20 eyes (27%) received a second FAc injection after a mean of 36.5 months (range 8.0–75.0). Remarkably, six eyes (8.1%) underwent reinjection with three or more FAc implants. A total of 26 eyes (35.1%) required additional treatment after a mean of 15.38 months (range 3.0–56.0). Of these, 11 eyes (14.8%) received additional intravitreal steroid treatment (mean 1.9 injections; range 1–5), and 20 eyes (27%) received intravitreal anti-VEGF therapy (mean, 15.6 injections; range 1–54).

Changes in IOP before and after FAc implantation in the overall cohort are shown in Fig. 3. Paired analysis revealed no statistically significant difference between baseline and follow-up IOP values (Wilcoxon matched-pairs signed-rank test; *p* = 0.364). Among 73 paired observations, 10 tied pairs were excluded, and the median IOP change was + 1 mmHg. 35 eyes, 14 (40%) required cataract surgery. No other implant-associated adverse events were observed.

## Discussion

In this real-world single-center cohort, the intravitreal FAc implantation produced a robust anatomical response and a modest overall improvement in BCVA. These outcomes are broadly consistent with both the pivotal randomized controlled trials and pooled real-world analyses of the FAc implant.

The FAME randomized trials demonstrated sustained CRT reduction and a durable visual benefit in chronic DME treated with the fluocinolone implant over three years, establishing the mechanistic and clinical rationale for long-acting intravitreal corticosteroid therapy in patients with persistent DME. Our anatomical results mirror the magnitude and durability reported in FAME and subsequent clinical trial follow-ups, supporting reproducibility of effect outside the tightly controlled trial setting [15]. 

Large real-world series and systematic reviews have reported mean peak visual gains around ~ 8 letters and mean CRT reductions of approximately 30–35% following FAc implantation, with variability according to baseline characteristics and chronicity of DME. The reductions in CRT in our cohort (median changes up to ~-75 μm overall and larger drops in selected subgroups) and the proportion of eyes demonstrating functional stabilization or improvement are therefore comparable to the central tendency of previously published real-world data. This concordance strengthens confidence that the FAc implant produces reproducible anatomical benefit in heterogeneous clinical practice populations [19]. 

Our subgroup analyses demonstrated greater CRT reduction, better visual outcomes and longer treatment durability in eyes with limited prior anti-VEGF exposure compared with eyes that had received six or more injections. In addition, the number of prior anti-VEGF injections was negatively correlated with subsequent treatment durability after FAc implantation. These findings align with the clinical observation, reported in multiple real-world series, that earlier use of sustained-release steroid implants in the disease course can be associated with longer intervals to retreatment and lower procedural burden, whereas prolonged anti-VEGF exposure frequently characterizes eyes with more chronic, treatment-resistant DME [20, 24, 25]. Notably, chronicity and cumulative treatment burden - often reflected by multiple prior anti-VEGF injections - may cause retinal stretching and potentially irreversible damage [26] and are linked to reduced responsiveness to subsequent interventions, a pattern that our data reproduce [27]. 

By contrast, prior short-acting intravitreal corticosteroid exposure did not abolish response to the FAc implant in our cohort: although eyes with prior corticosteroid injections showed somewhat smaller median CRT reduction, they nonetheless experienced statistically significant anatomical benefit and no clear reduction in durability. This observation is consistent with published reports indicating that prior corticosteroid responsiveness is not a prerequisite for benefit from sustained-release corticosteroids, though many regulatory labels (and clinical practice patterns) favor implantation in patients who previously tolerated steroids without significant IOP rises [27]. 

The dissociation between marked CRT reduction and only modest BCVA improvement, particularly among eyes with extensive prior anti-VEGF treatment or chronic edema, was a prominent finding in our series. This structure–function dissociation has been repeatedly observed in both trials and clinic cohorts and is commonly attributed to irreversible retinal damage (photoreceptor loss, disorganization of retinal inner layers, chronic ischemic change) that persists despite resolution of intraretinal fluid [28, 29]. Our functional data therefore reinforce the notion that anatomic drying is necessary but not always sufficient to restore vision in longstanding DME. Clinicians should counsel patients with chronic or heavily pretreated DME that anatomical improvement may not translate into large visual gains [15]. 

We did not observe a statistically significant change in IOP in paired analyses, but interpretation is limited by non-standardized documentation of IOP-lowering interventions during follow-up. Interpretation of these findings is limited by the lack of systematic assessment or adjustment for IOP–lowering medications or surgeries during follow-up, which may have influenced measured IOP values. This echoes the balanced safety profile reported in many real-world series: while steroid-related IOP increases and cataract progression are well documented risks [9] (and remain the principal safety concerns with FAc), rates and clinical consequences vary across cohorts and appear manageable with routine monitoring and standard IOP-lowering measures in selected patients. Our findings therefore reinforce the need for individualized risk–benefit assessment and regular IOP surveillance after implantation.

The retrospective design of this study led to several limitations. Treatment decisions were heterogeneous and documentation of prior therapies, disease duration and follow-up was incomplete. Consequently, the patient population was heterogeneous in terms of retinopathy severity and responsiveness to the FAc implant, which partly limited the ability to demonstrate statistical significance.

One important limitation of this study relates to potential selection bias in the timing of the FAc implant. The number of prior anti-VEGF injections was not determined by a standardized treatment protocol but rather reflected real-world clinical decision-making.

In our clinical setting, multiple physicians were involved in planning and administering intravitreal therapy, and each clinician followed their own therapeutic approach regarding the choice and sequencing of treatment, including anti-VEGF agents and corticosteroids. As a result, the decision of when to switch to the FAc implant varied between physicians and patients. While this introduces variability and potential confounding, it also reflects real-world clinical practice, which may increase the generalizability of our findings to routine care settings.

Assessment of the inner and outer retinal layer integrity was not consistently feasible, as in some cases only OCT printouts rather than full 3D volumetric scans were available. Therefore, the influence of photoreceptor and outer retinal layer status on clinical outcomes could not be reliably evaluated.

Prospective studies are warranted to refine patient selection and to clarify the positioning of corticosteroid therapy within the treatment algorithm in order to maximize anatomical and functional outcomes.

To conclude, this real-world study demonstrates FAc implantation provides a sustained and robust anatomical benefit in eyes with DME, as evidenced by significant and durable CRT reduction across the overall cohort and predefined subgroups. Functional outcomes showed a modest but statistically significant improvement in BCVA in the overall population, although visual gains were not significant in heavily pretreated eyes, reflecting substantial inter-individual variability. Treatment durability was strongly influenced by prior therapy, with earlier use of the FAc implant, particularly before extensive anti-VEGF exposure, associated with longer therapeutic effect and reduced need for additional interventions. Despite limitations inherent to the retrospective design, heterogeneous treatment decisions, and incomplete systemic data, these findings support the FAc implant is an effective long-term treatment option for DME.

## Data Availability

All data generated or analyzed during this study are included in this article. Further enquiries can be directed to the corresponding author.

## Abbreviations

FAc: Fluocinolone acetonide
DME: Diabetic macular edema
anti-VEGF: Anti–vascular endothelial growth factor
BCVA: Best-corrected visual acuity
CRT: Central retinal thickness
IOP: Intraocular pressure
IQR: Interquartile range
